# Notes and News

**Published:** 1898-01-22

**Authors:** 


### NOTES AND NEWS.

(COLLECTED AND COMMUNICATED.)

THE Local Government Board have sanctioned an expenditure by the Metropolitan Asylums Board of £227,[illegible] for the erection of the proposed Joyce Green Small-pox Hospital.

THE annual meeting of the After-Care Association for Poor Persons Discharged Recovered from Asylums for the Insane will be held on January 31st, 1898, by the kindness of Sir William and Lady Broadbent, at 84, Brook Street, Grosvenor Square, W.

THE people of Coventry have expended £1,500 upon their hospital—the Coventry and Warwickshire Hospital—as a Jubilee offering. The interior of the building has been repaired, redecorated, and altered. Eight new beds have been added, new drains laid down, and sanitary appliances provided, whilst the laundry has been refitted and altered.

A CONVERSAZIONE was held in the Charing Cross Medical School on the 10th inst. Most of the medical staff and students were present, and a variety of entertainments were provided for the numerous guests, amongst which were lectures, exhibitions of curios from the Far East, demonstrations of the x-rays and wireless telegraphy, musical and dramatical performances.

THE death of Dr. James Cagney, and the resignation of Mr. Pearce Gould, owing to pressure of work elsewhere, have given rise to the following appointments at the Hospital for Epilepsy and Paralysis: W. Aldren Turner, M.D., F.R.C.P., as physician in charge of in-patients; H. Campbell Thomson, M.D., M.R.C.P., physician in charge of out-patients and registrar; A. Pearce Gould, F.R.C.S., consulting surgeon; and G. Lenthal Cheatte, surgeon.

THE Archbishop of Canterbury addressed the students of the London Hospital on the opening of the winter session. The address was admirable, and the departure from the usual plan of securing the services of some exponent of medicine was a happy one. The ideal placed before students of any branch of learning can never be too high, and such a man as the Archbishop of Canterbury is well able to point out with authority the special attributes, apart from the scientific, which go to make the ideal physician.

MR. MICHELLI, secretary of the Seamen's Hospital Society, is going to Egypt on sick leave, sailing in the P. and O. ss. *Sunda*, on Friday, the 21st inst.

THE foundation-stone of a new isolation hospital for the Croydon district has been laid at Beddington Corner. Three pavilions are to be provided, for scarlet fever, diphtheria, and for patients under observation. The total number of beds will be 28. The usual offices and accommodation for the staff, mortuary, and post-mortem room complete the buildings, which are estimated to cost £15,878.

BEDFORD is certainly in a dangerous plight at present. It appears that the infirmary governors gave notice that they were about to close the old fever hospital administered by them, but the local authorities took no steps to provide a substitute, and consequently Bedford and the surrounding district is in the dangerous position of having no harbour for fever patients now that the infirmary governors have closed their building. A petition is on foot to keep the old hospital open until a new one is provided.

THE anonymous gift of £25,000 to the London Hospital is accompanied by two conditions—the one being that the out-patients attending the out-patients' department, upon the erection of which the money is to be expended, shall pay a small fee towards the cost of medicine and appliances; and the other that letters of admission shall be abolished. The hospital has received another gift of considerable amount since, and also £8,937 from the Prince of Wales's Fund, which must help to remove some of the anxiety felt by the authorities in providing for the wants of the great hospital.

THE Royal Photographic Society is organising an exhibition at the Crystal Palace on April 27th. The scientific application of photography is to be fully demonstrated, and the X ray photographs will play a large part. The section devoted to scientific photography will include photo-micrographic apparatus, photographic recording instruments, spectroscopic photography, and examples of the application of photography and of apparatus in connection with nearly all the sciences. Our readers who may be able to lend any examples of the application of photography to medicine and surgery are invited to communicate with the assistant-secretary at the society's rooms, 12, Hanover Square.

THE question of what will prove of interest or not in a hospital report is always a difficult problem. Some secretaries are very happy in the manner in which they place the events of the hospital year before subscribers and readers of the report, whilst others make it appear that the year has been a period of uneventful routine. We are always very glad to bring to notice any document which strikes us as bearing the mark of originality, and as likely to attract interest. Some leaflets from the Royal Hospital, Richmond, have reached us, and on one of them is set forth the actual number of patients coming from each district. Now this is an excellent idea; it helps to point out where responsibility lies to recognise benefits received, and as a district grows indicates where outpost hospitals are needed. It is often pointed out that support is not always coming from the direction whence most patients are drawn, but in very few instances is it clearly brought to the notice of those interested in the work of a hospital.

